# Gasotransmitter ammonia accelerates seed germination, seedling growth, and thermotolerance acquirement in maize

**DOI:** 10.1080/15592324.2022.2163338

**Published:** 2023-01-22

**Authors:** Zhong-Guang Li, Xiao-Qiong Lu, Ji Chen

**Affiliations:** School of Life Sciences, Engineering Research Center of Sustainable Development and Utilization of Biomass Energy, Ministry of Education, Key Laboratory of Biomass Energy and Environmental Biotechnology, Yunnan Normal University, Kunming, P.R. China

**Keywords:** Ammonia, seed germination, seedling growth, seedling thermotolerance, maize

## Abstract

Ammonia (NH_3_), as an intermediate product of nitrogen metabolism, is recognized as a novel gasotransmitter (namely gaseous signaling molecule), its signaling role being revealed in plants. NH_3_ exists in two different chemical forms, namely the weak base (free molecule: NH_3_) and the weak acid (ammonium: NH_4_^+^), which are generally in equilibrium with each other in plants. However, the effect of NH_3_ on seed germination, seedling growth, and thermotolerance acquirement in maize remains unclear. Here, maize seeds were imbibed in the different concentrations of NH_3_·H_2_O (NH_3_ donor), and then germinated and calculated seed germination rate at the various time points. Also, the 60-h-old seedlings were irrigated in the different concentrations of NH_3_·H_2_O, and then subjected to heat stress and counted survival rate. The data implied that the appropriate concentrations (6, 9, and 12 mM) of NH_3_·H_2_O accelerated seed germination as well as increased seedling height and root length compared with the control without NH_3_ treatment. Also, the suitable concentrations (2 and 4 mM) of NH_3_·H_2_O improved tissue vitality, relieved an increase in malondialdehyde content, and enhanced survival rate of maize seedlings under heat stress compared with the control. These results firstly suggest that NH_3_ could accelerate seed germination, seedling growth, and thermotolerance acquirement in maize.

## Text

Ammonia (NH_3_) not only is an intermediate product of nitrogen metabolism but also a novel gasotransmitter, namely gaseous signaling molecule, in plants.^1^ NH_3_ exists in two different chemical forms, namely the weak base (free molecule: NH_3_) and the weak acid (ammonium: NH_4_^+^), which are generally in equilibrium with each other in plants.^2^ Therefore, NH_3_ may be exerted its biological effects in the forms of weak base (NH_3_) and/or weak acid (NH_4_^+^) forms, similar to hydrogen sulfide (both H_2_S and HS^−^ forms). NH_3_, similar to other gaostransmitters H_2_S, nitric oxide (NO), carbon monoxide (CO), ethylene (ETH), methane (CH_4_), hydrogen gas (H_2_), sulfur dioxide (SO_2_), and hydrogen cyanide (HCN), plays a crucial physiological role in plant growth, development, and response to environmental stresses.^1^ Meanwhile, NH_3_ exhibits a dual role during performing its physiological processes, that is, as gasotransmitter at low concentration and toxic agent at high dosage.^3,4^ Therefore, NH_3_, the same as signaling molecules calcium ion (Ca^2+^), hydrogen peroxide (H_2_O_2_), and other gasotransmitters, must be maintained homeostasis in plant cells. The homeostasis of NH_3_ in plant cells is minutely governed by its production, transport, and scavenging via enzymatic and non-enzymatic pathways, such as nitrate reduction, photorespiration, amino acid catabolism, and phenylpropanoid metabolism.^1,5^ Similar to other signaling molecules, NH_3_ signaling can be triggered when its active production exceeds scavenging, and then cascades downstream signaling pathways, thus regulating many physiological processes in plants.^6,7^

NH_3_, as gasotransmitter, participates in plant growth, development, and response to adverse environmental stresses. NH_3_ could inhibit the elongation of primary and lateral roots by crosstalk with pH and reactive oxygen species (ROS) signaling pathways, promotes lateral root branching by auxin signaling pathways, and regulates root hair development by signaling network involved in Ca^2+^, ROS, and pH oscillations.^1,3^ Similarly, in rice seedlings, NH_3_ could enhance drought tolerance by interplaying with abscisic acid and aquaporins via facilitating water uptake by roots.^5^ Also, NH_3_ was able to increase the resistance of tomato plants to pathogen infection by triggering ROS burst.^8^ However, in maize, the effect of NH_3_ on seed germination, seedling growth, and thermotolerance acquirement remains indistinct.

To investigate the effect of NH_3_ on seed germination, seedling growth, and thermotolerance acquirement of maize (*Zea mays* L.), the maize (Qiuqing No 88) seeds with the same size and health were sterilized in 5% (v/v) sodium hypochlorite solution for 15 min, and then washed cleanly and imbibed in the different concentrations (0, 3, 6, 9, 12, and 15 mM) of ammonia water (NH_3_·H_2_O) for 12 h. Afterward, the soaked seeds were sown on the wetted filter paper (with six-layer) in culture bottles and germinated at 26°C in the dark in climatic chamber. Germination rate was calculated at the indicated time points. Similarly, the soaked seeds by distilled water were germinated at 26°C for 60 h in the dark in climatic chamber. The 60-h-old seedlings were irrigated with 100 mL of the different concentrations (0, 2, 4, and 6 mM) of NH_3_·H_2_O for 4 h, and then rinsed in 100 mL of distilled water to remove NH_3_ residue. The control seedlings (irrigated with distilled water) and NH_3_-treated seedlings were transferred into another climatic chamber with 47°C in the dark for 16 h to subject heat stress. After heat stress, the heated seedlings were recovered at 26°C, 100 μmol m^−2^ s^−1^, and 16/8 h (light/dark) photoperiodism for a week in climatic chamber. Finally, the survival rate was counted according to the previous methods.^9^ Experiments were repeated at least three times, and the data was assessed as per one-way analysis of variance and the least significant difference. Asterisk (*) and double asterisks (**) in figures represent significant difference (*P* < .05) and very significant difference (*P* < .01) compared with the control without NH_3._H_2_O treatment, respectively.

**Figure 1.**
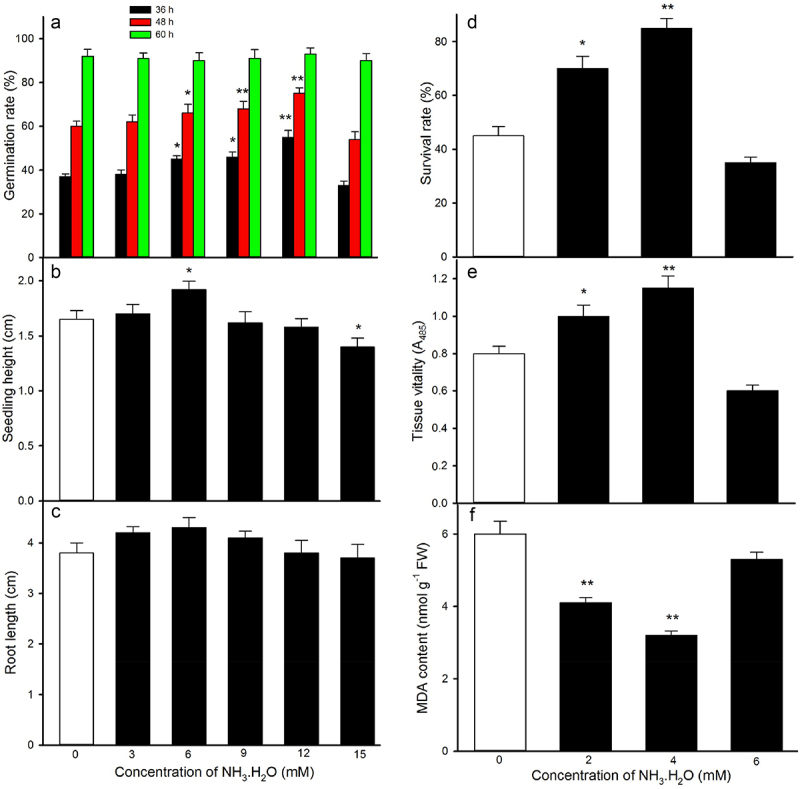
Effect of ammonia water (NH_3_⋅H_2_O) on seed germination (a), seedling growth (b, c), and thermotolerance acquirement (D, E, F) in maize. Data in figure represent the mean ± SE (n = 3), significant difference (*P* < .05) and very significant difference (*P* < .01) are indicated by asterisk (*) and double asterisks (**) compared with the control without NH_3_.H_2_O treatment, respectively.

The results show that seed-imbibition with the different concentrations of NH_3_·H_2_O accelerated seed germination as well as increased seedling height and root length in a concentration-dependent manner. The germination rate of seeds imbibed with 3, 6, 9, 12, and 15 mM reached 38% (62% and 92%), 45% (*P*˂ 0.05) [66% (*P*˂ 0.05) and 90%], 46% (*P*˂ 0.05) [68% (*P*˂ 0.01) and 91%], 55% (*P*˂ 0.01) [75% (*P*˂ 0.05) and 93%], and 33% (54% and 90%) at 36 h (48 and 60 h), respectively, compared with the control 37% (60% and 92%) (Fig1. A, supplement). Among the concentrations, the concentration of 12 mM was the most effective concentration for the germination of maize seeds (Fig1. A, supplement). For seedling growth, the seed-imbibition with 6 mM significantly increased the height of maize seedlings (*P*˂ 0.05), but other concentrations (3, 9, 12, and 15 mM) had no significant effect on seedling height and root length compared with the control (Fig1. B, C, supplement). Similarly, the root-irrigation with the different concentrations of NH_3_·H_2_O enhanced survival rate (Fig1. D, supplement) and tissue vitality (Figure 1. E), as well as relieved an increase malondialdehyde content (Figure 1. F) in maize seedlings under heat stress in a concentration-dependent manner. The survival rate, tissue vitality, and malondialdehyde content of maize seedlings irrigated with 2, 4, and 6 mM were 70% (1.0 A_485_ and 4.1 nmol g^−1^ fresh weight FW) (*P*˂ 0.05), 85% (1.15 A_485_ and 3.2 nmol g^−1^ FW) (*P*˂ 0.01), and 35% (0.6 A_485_ and 5.3 nmol g^−1^ FW), respectively, compared with the control 45% (0.8 A_485_ and 6 nmol g^−1^ FW) (Figure 1. D, E, F). Among the concentrations, the concentration of 4 mM was the most effective concentration for thermotolerance acquirement in maize seedling (Figure 1. D, E, F). These data illustrate that NH_3_ could accelerate seed germination, seedling growth, and thermotolerance acquirement in maize. The seed germination, seedling growth, and thermotolerance acquirement are complex physiological processes, which are involved in plant hormones, antioxidant system, osmotic adjustment system, ion balance system, heat shock proteins, and so forth.^10^ Patterson et al.^7^ reported that NH_3_ could trigger ROS signaling and activate ROS-scavenging system in Arabidopsis plants. Similarly, NH_3_ could regulate the growth of roots and root hairs as well as improve drought tolerance by the crosstalk among Ca^2+^, ROS, pH, and phytohormones (abscisic acid and auxin).^3,5^ Also, NH_3_ was able to improve salt tolerance in citrus plants by modulating abscisic acid, hydrogen peroxide, polyamines, and proline.^11,12^ However, the underlying mechanism of NH_3_-accelerated seed germination, seedling growth, and thermotolerance acquirement needs to be further explored in the coming days.

## Supplementary Material

Supplemental Material
