# Supplementary material for: Gasotransmitter ammonia accelerates seed germination, seedling growth, and thermotolerance acquirement in maize
Source: Plant Signal Behav. 2023 Jan 22;18(1):2163338. doi: 10.1080/15592324.2022.2163338 (PMC9869984; doi:10.1080/15592324.2022.2163338)
Supplement: Supplemental Material [file KPSB_A_2163338_SM7072.pdf]

Supplement

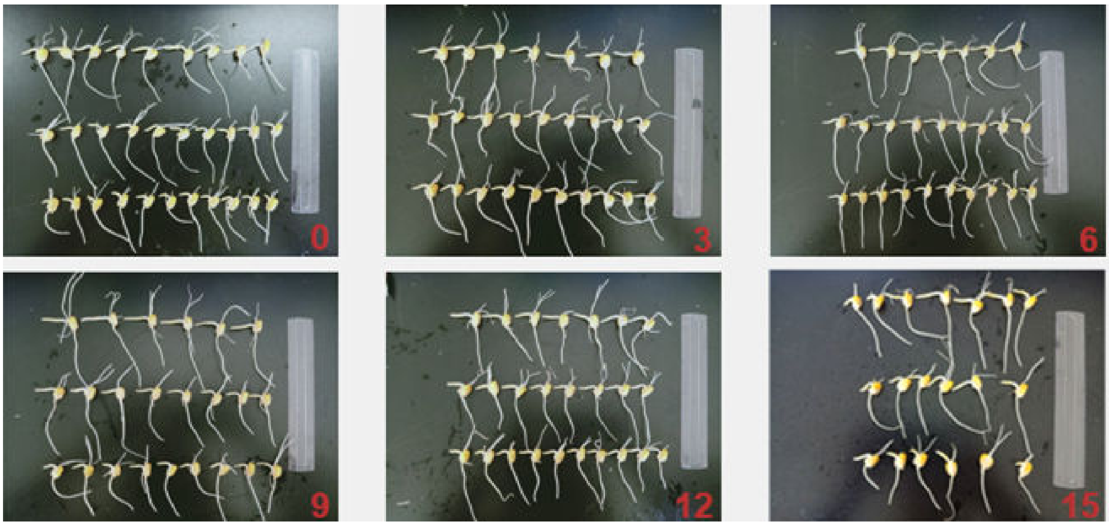

Effect of the different concentrations (0, 3, 6, 9, 12, and 15 mM) of $NH_3.H_2O$ on seed germination and seedling growth of maize.

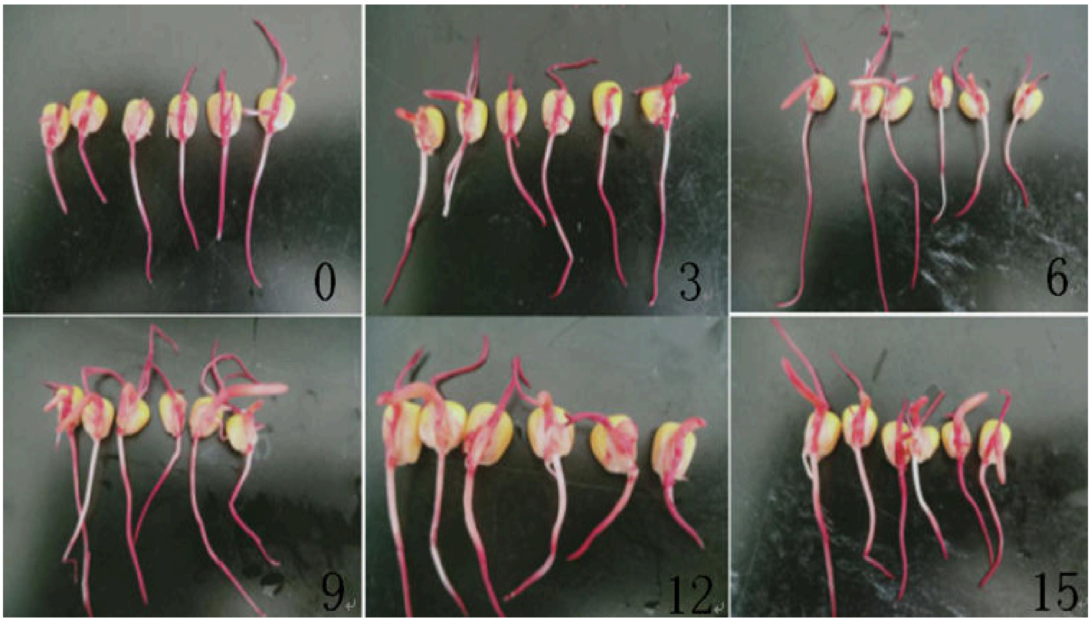

Effect of the different concentrations (0, 3, 6, 9, 12, and 15 mM) of $NH_3.H_2O$ on seedling vitality.

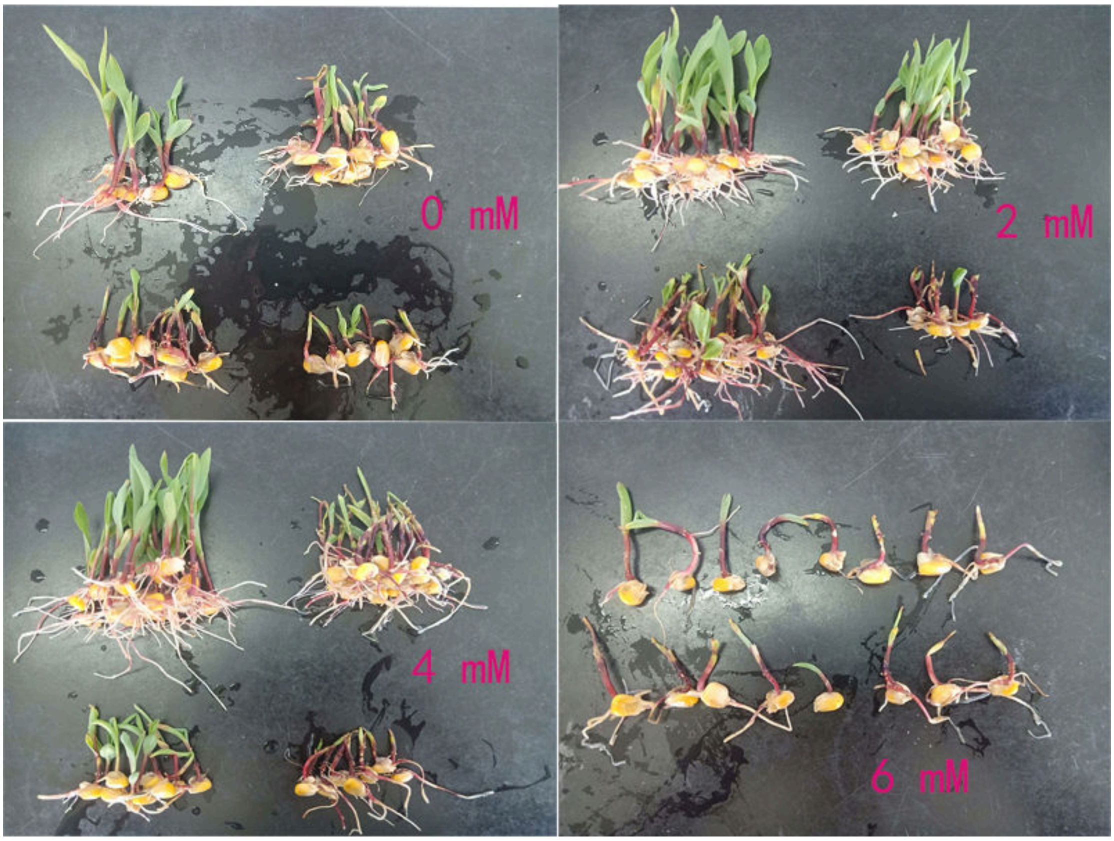

Effect of the different concentrations (0, 2, 4, and 6 mM) of $NH_3.H_2O$ on survival and regrowth of maize seedlings after high temperature.
